# How Intergenerational Injustice Impacts Young People's Mental Health: A Qualitative Study

**DOI:** 10.1111/eip.70273

**Published:** 2026-10-04

**Authors:** Gerald Jordan, Sophie Webb, Beckye Williams, Catrin Street‐Mattox, Emma Černis, Stephanie Burnett‐Heyes

**Affiliations:** ^1^ University of Bristol, School Psychological Science Bristol UK; ^2^ University of Birmingham, School of Psychology, College of Life and Environmental Science Birmingham UK

**Keywords:** intergenerational injustice, social determinants of health, youth mental health

## Abstract

**Introduction:**

Emerging adulthood is a significant period of developmental transition, and recent increases have been observed in mental health diagnoses and service use among people at this stage of life. Coinciding with these increases is a perceived decrease in young people's quality of life across advanced economies such as the UK, on account of rising economic, social and political precarity. The disparity between the relative stability experienced by previous generations and the instability with which younger generations contend is known in policy spaces as ‘intergenerational injustice’. Despite its importance, no study has examined how intergenerational disparities are subjectively impacting young people's mental health. To address this gap, our study sought to understand (1) *how intergenerational injustice is subjectively experienced by young people in the UK? and* (2) *how intergenerational inequities impact young people's mental health.*

**Methods:**

A qualitative descriptive approach was used. 18 young people participated in semi‐structured interviews addressing the research questions. Interviews were transcribed verbatim and analysed using reflexive thematic analysis to generate themes and patterns from the data.

**Results:**

Our analysis generated three interrelated themes: (1) political abandonment, (2) greater precarity around the means to support one's existence and potential, and (3) limited opportunities for living a full, healthy life.

**Discussion:**

This is the first qualitative study to explore how intergenerational injustice subjectively impacts young people's lives and mental health. Governments, policymakers, and researchers should better address intergenerational inequalities, given the importance of emerging adulthood for life trajectories, including mental health diagnoses.

## 1 Introduction

Emerging adulthood spans 18–29 years of age, marking a time of significant transition in people's educational, occupational and familial life (Arnett 2000) and a period of increased susceptibility to mental health problems for both neurological and sociocultural reasons (Patel et al. 2021; Sisk and Gee 2025). The onset of mental health problems typically peaks in emerging adulthood and often disrupts developmental processes which are integral to healthy progression to adulthood (Solmi et al. 2022).

A recent increase in mental health diagnoses and service use among emerging adults has been observed in advanced economies (Armocida et al. 2022). Contributing factors to this rise may include exponential increases in social media use (Orben et al. 2024), as well of isolation resulting experiencing social isolation during from COVID‐19 lockdowns (Wolf and Schmitz 2024).

Of note, younger generations in advanced economies such as the UK are now worse‐off than both their parents' and grandparents' generations across several key social determinants of health (Christophers 2018). These include living in increasingly unaffordable and insecure housing (Alidoust and Huang 2023), the rise of precarious work for limited wages (Puig‐Barrachina et al. 2014), increased debt (Sinha et al. 2024) and the growing impacts of climate change on health (Martin 2024). Widening inequities between generations have been referred to as ‘intergenerational injustice’ in policymaking spaces (Kershaw et al. 2017).

Within political philosophy, Rawls (2001) developed an influential theory pertaining to the concept of justice and intergenerational justice. Rawls discussed what future generations are owed according to a just savings rate, where savings include resources that future generations will have access to regardless of whether current generations have planned for those resources to be provided or not. A just society is one in which all people are respectfully treated by considering their economic conditions, social relations and status, and politics; and a just society is built upon a fair distribution of resources, as well as relational, social and political equality (Galasso et al. 2025). In the context of intergenerational injustice, this perspective highlights that justice between generations is not limited to material resources alone, but also concerns whose interests may one day be recognised, whose voices may one day shape decision‐making, and whose life chances will be protected over time. Relatedly, Caney (2019) highlights how advanced economies are harming future generations by engaging in deterimental short‐termism whereby short‐term gains are prioritised over the wellbeing of generations to follow, undermining intergenerational justice.

The importance of intergenerational injustice has been highlighted in UK and international media discourse (Pickard 2019) and there have been efforts to address intergenerational injustice by certain UK governments (Future Generations Act 2015). The potential role of intergenerational injustice in harming young people's mental health was recently raised by the Lancet Commission on Youth Mental Health (McGorry et al. 2024) and in editorials (Jordan et al. 2024). However, there has been no exploration of how intergenerational injustice is subjectively experienced and understood by young people in the UK as emerging from their social, economic, political, and environmental conditions, and how these experiences shape their mental health. Addressing this knowledge gap can support policies and other initiatives to address the impact of intergenerational injustice on young people's mental health, in turn encouraging UK decision‐makers to adopt such policies; alert clinicians to the need to address these inequities within services; and give voice to younger generations who feel such inequities have influenced their mental health.

To address this gap, our study addressed two research questions: (1) *How is intergenerational injustice subjectively experienced by young people in the UK?* and (2) *How have intergenerational inequities impacted young people's mental health?*

## 2 Methods

### 2.1 Overall Approach

A social constructivist paradigm guided this study, acknowledging how the existence of multiple social realities may shape knowledge, data collection and analysis (Guba and Lincoln 2005). A qualitative descriptive methodology was used to collect and analyse data in a way that closely reflected participants' words and experiences, while allowing for rich interpretation that drew heavily from policy research (Sandelowski et al. 2007).

### 2.2 Setting and Participants

18 participants were recruited via electronic mental health listservs (e.g., National Survivors Union Network), thinktanks (e.g., the Intergenerational Foundation), social media (e.g., X) as well as through snowball and word of mouth sampling. Recruitment took place between 2022 and 2023, before the Labour government came to power. Participants must have been young adults aged 18–30; residing in the UK; fluent enough in English to provide informed consent and participate in an interview; and subjectively feel as though intergenerational injustice was impacting their mental health. Due to potential unfamiliarity with the concept, our recruitment materials stated: ‘do you feel you are worse off than your parents were when they were your age in terms of finances, housing and employment?’. Participants received a £20 Amazon gift voucher upon study completion. Participants were recruited until information power had been achieved (Malterud et al. 2015). Five items have been proposed to impact the information power of a sample, including the study aims, sample specificity, use of established theory, quality of dialogue, and analytic strategy. A study will require fewer participants with a highly specific aim, if it is grounded in theory, if the interview dialogue is strong, and if longitudinal analyses are used.

### 2.3 Data Collection

Online semi‐structured interviews (mean length = 40 min) were conducted by a trained research associate supervised by the first author. Our interview guide contained probes assessing participants' perceptions of government performance at addressing intergenerational injustice; generational differences in employment, housing, finance and the impacts of climate change; and how these inequities impacted participants' mental health. Our interviews were open to discussing additional elements related to the topic (e.g., social media) and our guide evolved with each subsequent interview. Reflections were noted after each interview and discussed with the first author. Automatically generated transcripts were checked for accuracy and subject to analysis.

### 2.4 Data Analysis

The first author conducted a reflexive thematic analysis using NVivo (v 1.4) (Braun and Clarke 2021). First, each transcript was read to gain an initial understanding of participants' experiences. He developed set of preliminary inductive codes reflecting what participants had said, as well as deductive codes drawn from relevant literature on intergenerational injustice and discussions with policy makers on the topic. He then mapped out preliminary patterns observed in the data. Transcripts were then subjected to one round of sentence‐by‐sentence coding to develop a rich dataset reflecting participants' experiences. Both deductive and inductive codes were applied, reflecting participants' own words and experiences relevant literature was reflected upon during this process to aid interpretation. Similar codes that answered our research questions were grouped into focused codes, sub‐themes, and themes that were in turn organised into an overarching narrative reflecting the totality of participants' accounts. Rigour was established by ensuring congruence between quotations, codes and themes. In addition, the interviewer and first author engaged in reflexive practice by discussing, making sense of, and understanding our positionality when analysing the data by examining the multiple brought selves we brought to the project (Reinharz 1997).

For instance, we reflected on our personal selves as people from different generations both impacted by precarious employment and living conditions at different life stages, and how we sometimes related to participants' own experiences. The first author reflected on being a newcomer to the UK. He and the interviewer (who was born in the UK) engaged in many rich conversations about the social, policy and economic landscape of the country, which informed the analysis. We also reflected on our own political orientation, and how to honour what participants had said during the interviews even when, at times, we disagreed with participants' views on a topic.

## 3 Ethics

Ethical approval for the study was granted by the University of Birmingham Science, Technology, Engineering and Mathematics Ethical Review Committee (ERN_1030‐Sep2023). Prior to participating, participants read information sheets, signed consent forms, and were informed of UK‐based mental health support services for additional support.

## 4 Results

Participant characteristics are displayed in Table 1. Participants described how successive governments had abandoned young people, leading to a sense of disillusionment and social, financial and environmental insecurity that undermined the wellbeing of youth living in the UK. While political, social and economic factors were seen as the primary drivers of these intergenerational inequities, participants also highlighted the role of technology (e.g., social media) and the media in both harming and benefitting their lives. Additional quotations illustrating each theme are included in Table 2.

**TABLE 1:** Demographic characteristics of the sample.

Variable	*M*/SD; *f/%* (*n* = 18)
Age	24.59 (3.57)
Sex	
Female	10 (55.6%)
Male	8 (44.4%)
Gender	
Cis male	8 (50%)
Cis female	9 (44.4%)
Non‐binary	1 (5.6%)
Sexuality	
Straight	13 (72.2%)
Gay or lesbian	3 (16.7%)
Bisexual	2 (11.1%)
Education	
Undergraduate	7 (33.3%)
A‐levels	4 (19.1%)
Secondary	4 (18%)
Masters	3 (14.3%)
Yearly household income	
More than £34 000	10 (55%)
Less than £34 500	8 (44.4%)
Relationship status	
Single	10 (55.6%)
In one relationship	6 (33.3%)
In more than one relationship	1 (5.6%)
Legally married or civil partnership	1 (5.6%)
Ethnic or racial background	
Black British	4 (19%)
White British	3 (14.3%)
African	1 (4.8%)
Asian	1 (4.8%)
Black African	1 (4.8%)
British Pakistani	1 (4.8%)
Mixed race	1 (4.8%)
South Asian	1 (4.8%)
Sri Lankan	1 (4.8%)
Home status	
Renting your home	14 (77.8%)
Living rent free	3 (11.1%)
Living with parents and paying rent	1 (11.1%)
Employment status	
Employed part time	10 (58.8%)
Employed full time	5 (29.4%)
Prefer not to say	2 (11.8%)

**TABLE 2:** Participant quotations.

Theme	Subtheme	Quotation
Political abandonment		‘I just feel like, so what is this country doing for me? Because I do my part, I pay my taxes, I don't do anything illegal. And yet I'm just sort of like, and you know, there's nothing, I can't really do anything‐ if I go to sort of the money help advisors or Job Centre or whatever, they just like, yeah, nothing they can do. I apply for extra benefits but I'm not eligible. I'm kind of like, too rich to be poor, but not rich enough to be rich, if that makes sense? (P19)’
Greater precarity around the means to support one's existence and potential	Housing insecurity	‘There's a completely different comparison, and I think it's much worse, as compared to my parents' generation. Like, as I said, they've, my parents have savings like, they've bought their house, you know. They were able to buy another house as well. So they have two houses already which have been paid off, that have no mortgages to pay. So I think they're in a good position, whereas myself, like, I've been renting where I live. I don't have, you know, income enough even from the work I do. Have to pay for rent, I have to pay for, for extras like gas, electricity, food. Everything, like cleaning utilities. So I think, it's a complete difference now, because, first of all, the work does not pay enough in order to, to get by. And on top of that, the biggest problem is cost of living crisis. (P5)’
	Insecurity around education	‘My husband, he's 31. But he was told, go to university and you'll definitely get a good job. But middle management marketing jobs are all taken up. They're all filled up like, there are no more. So, you know, his degree kind of feels useless. And I know lots of people whose degrees feel useless to them, because there simply wasn't space in the sector, or there was no progression, or the pay was actually rubbish, or whatever (P6)’
	Income precarity	‘I think that I have more, that I have more debts than my parents, because at this time I had to acquire some loans, student loans. And I'm, I'm also supposed to pay my rent. So I think I'm in a tougher situation. (P10)’
	Employment precarity	‘Also jobs, employment. Like, you could work without a university degree. Like, I know one of my friends, her dad's a lawyer, and he's, he's quite old, he's like, in his seventies. But he's a lawyer, he does not‐ he didn't go to law school. But he is a lawyer and a practising lawyer, and like, he just did a course and he's fine, and I feel like there's so much of that. And now it's like insane like, even in research, you can't do like, unless you have a PhD. Which, by the way, is like, ten years of education. For getting paid, what? £35 000 a year? I'm sorry (P13)’
	Environmental insecurity	‘I do think like, global warming and all of that, I do think nature is a bit like, we're at the worst the Earth has been physically like, in terms of nature. I do think that our generation is struggling a lot in terms of quality of everything, like the water that we drink, just because our water sources are polluted now. Or you know, with deforestation and all of these things, I do think we're worse off. And the same thing, for like, trash. I mean, if I think about where our stuff goes, it gives me so much anxiety because I'm like, I don't know. I know I consume so, I consume food and clothes, and when I give those away, or if I have to throw something. I can't even imagine how much waste I'm accumulating, let alone like, seven billion people. And where does that go? (P13)’
Limited potential for living of a full, healthy life	Poorer mental and physical health	‘All the situations, you're always thinking about the rent, on a monthly basis. You know, how to cover the rent and feeding. It's always something that brings about anxiety, you know, worries about where to source the funds to, you know, take care of this important aspect of life because I feel like having‐ where to stay, it's a very big, important aspect of life. And this is one of the priorities in life. And we still have the depression, anxiety, and all sort of mental stuff like that. And I feel like it also has an impact on me because, you know, like I said earlier, I'm struggling financially. So I'm not very stable. I'm not stable. So this definitely makes me think a lot, makes me feel like, yeah, I‐ you know, things like that (P16)’
	Reduced opportunities for independence and self‐determination	‘I came to uni deciding to live at home because, financially, that was the more responsible thing to do. And then I kind of realised like, realised that it, the culture of moving out is really important and like, you know. It's sad how good it is for someone's like, personal development to move out. And then they move up to their peers and kind of cook for themselves, and really get like, find their own routine. You know, you're kind of working your way through that independence, but simultaneously having that cushion of a university… And so kind of being less independent and living at home definitely affected my sense of belonging, making friends and stuff. (P18)’

### 4.1 Theme: Political Abandonment

Participants felt that the needs of youth have increasingly been abandoned and not prioritised by successive governments. They pointed to major political initiatives and policies—such as Brexit and raising tuition fees—as failing to consider the needs of younger generations, leading to a sense of political abandonment, a loss of political efficacy, and diminished hope that any government would truly support them.

Compared to previous generations, participants felt that today's youth possess weaker political power, highlighting the lack of young people—particularly youth of colour—within government. Moreover, participants argued that governments have failed to adequately fund services of importance for youth (e.g., mental health services, affordable housing such as council housing).

Participants believed the government had been slow, or had failed to address growing economic and social precarity facing young people. They also felt that initiatives which they care about (e.g., aimed at tackling climate change) were largely inadequate and performative. By contrast, participants perceived that policies are increasingly geared towards supporting the governing and upper class—key voters—rather than middle‐ and working‐class youth. Upon reflecting on the general performance of the government towards young people, one participant reflected:

In my opinion, I feel like they have abandoned the young. Their [young] generation, compared to their older generation. (P8)

### 4.2 Theme: Greater Precarity Around the Means to Support One's Existence and Potential

Most participants felt they required greater educational attainment than their parents to access the same employment opportunities; however, the benefits of education, often emphasised by their parents, were perceived to have never materialised. Many participants noted that, despite incurring debt for their degrees, their education felt ‘useless’ and rarely led to employment matching their qualifications. However, other participants, who were the first in their families to attend university, felt they had greater educational and employment opportunities.

Participants also felt less able to find reliable employment which offered stable working patterns and predictable salaries. Participants perceived that competition for jobs had intensified, even for entry‐level roles, while the availability of high‐paying positions had decreased. Participants felt that pressure to excel at work was greater than it was for previous generations; a pressure compounded by toxic workplace environments, lack of loyalty towards employees, and blurring of work‐life boundaries due to constant connectivity. One participant felt the opposite; that there were now greater protections for workers. Many participants also faced pressure to constantly achieve and continuously improve themselves. Participants felt that this pressure was again exacerbated by technology, most notably social media, smartphones, and work‐from‐home policies introduced during the COVID‐19 pandemic.

Wages were seen as failing to keep pace with the rising cost of living. Reductions in wage growth were seen as leading to a decline in living standards and an inability to afford the same goods and services previous generations could, including both resources fundamental for survival (e.g., food, heating) and leisure activities (e.g., dining out, holidays). Relatedly, most participants described having fewer savings and assets relative to previous generations alongside greater debt, particularly from student loans. Some participants whose parents were born outside the UK felt the opposite was true. Two participants expressed that previous generations enjoyed greater financial support from friends, families and communities. One participant feared that AI may drive further employment precarity for younger generations.

Participants described how the natural environment had declined over generations. Many noted a decline in snowfall, more frequent storms, hotter summers and wildfires; changes that contributed, for some participants, to decisions not to have children. Some expressed concern about worsening air and water pollution, as well as declining quality of outdoor spaces (e.g., parks) over time. Some participants felt that young people now prefer indoor, technology‐based socialisation over outdoor activities favoured by previous generations.

Finally, employment precarity impacted young people's housing experiences. All except one participant—whose parents were immigrants to the UK—felt that renting or purchasing a home had become more unaffordable, especially in the south of England. Participants felt that their parents' generation could comfortably live in cities where they were employed, whereas housing choices for younger people were more restrictive. Most participants rented in the private sector and remarked that, at their age, their parents already had a mortgage. Participants described how private renting was characterised by frequent rent increases and eviction fears, as well as difficulties affording rent. Some described feelings of dehumanisation, as their landlord's permission was required to make their accommodation feel more like home (e.g., hanging pictures on the wall), while others experienced discrimination when trying to rent (e.g., racism). Some participants lived with their parents to manage costs. Such experiences of employment precarity were highlighted by one participant:

Things like taking maternity leave and taking leave after you've had babies. My mom took a career break after having me and my sister or left or went part‐time fully. I work seven jobs. And my partner works part‐time. So, we kind of flipped it. But it's, but there's still not enough to put in savings. (P5)

### 4.3 Theme: Limited Opportunities for Living of a Full, Healthy Life

Participants overwhelmingly felt as though their generation had fewer opportunities to live a full, healthy life enjoyed by previous generations, and that the promise of a good life in the UK had collapsed for them and their peers. While some participants spoke generally about the mental health impact of these inequities, many provided more specific accounts of how these inequities led to high levels of stress, anxiety, depression, and sleep disturbances (e.g., nightmares), as well as a strong fear—and paralysis—over the future and what will come. Many felt that their economic conditions led to profound fear, insecurity, vulnerability, hopelessness and helplessness, with others feeling a sense of degradation. Experiences around housing precarity—particularly around the fear of eviction and unaffordable rent increases—led to profound anxiety among most participants. Experiences of financial insecurity, unaffordability, and uncertainty over having enough money to pay for bills each month were seen as major contributor to participants' feelings of anxiety and depression. Climate change and other natural disasters contributed to heightened levels of climate anxiety, feelings of paralysis, impending doom, and avoidance of the news. Several participants described how social media has led to feelings of competition and pressure leading to feelings of low self‐worth, inadequacy, disordered eating and bullying not faced by previous generations.

Additionally, some felt that young people's physical health had declined due to increased pollution, time pressures, food insecurity, lifestyle changes (e.g., video games, modes of transit), and among one second‐generation immigrant youth, eating less healthy foods. At the same time, participants also remarked that accessing mental health and wellbeing services seemed more difficult than for previous generations.

Finally, many also felt that economic uncertainty had delayed their ability to reach key milestones related to independence, such as having children, moving out of their family home, and learning important life skills (e.g., cooking for oneself). For instance, the impact of financial precarity on both mental health and personal development was highlighted by one mother, who expressed:

I have had very bad periods of mental health before, where I've really worried about money. I've really worried about the future for my children and how different it's gonna look. I've worried about having them without really thinking too much about it. And now, being in a situation where‐ we are very, we're still very lucky, if I, like I say, if I got in trouble, my parents would help me. But I don't think I will be able to do that for my kids, and that makes me feel really shit. (P6)

## 5 Discussion

The objective of this study was to understand how intergenerational injustice is subjectively experienced among youth living in the UK and how such injustice impacts young people's mental health. Our analysis generated three interrelated themes: *political abandonment*; *greater precarity around the means to support one's existence and potential*; and a *l* *imited potential for living a full, healthy life*. Our study is the first to highlight the significant impact that intergenerational injustice has on young people's mental health. Our findings lay an important empirical foundation for future research and policy work aimed at supporting the social, economic, and political lives of young people both in the UK and internationally. This paper highlights the urgent need for governments, health systems and services to address inequities that have emerged between younger and older generations, and which potentially drive poor mental health and increased service use.

Our findings are consistent with evidence demonstrating that concentrations of wealth, opportunities and assets are becoming inaccessible to young people relative to previous generations that emerged into adulthood when political and economic conditions were (in some ways and for some groups) more favourable to their development and wellbeing. Our findings are consistent with Caney's (2019) position that governments prioritise short term priorities over the needs of future generations, perpetuating harm. Despite these inequities, government priorities across advanced economies, including the UK, have generally favoured the needs of older people rather than supporting the needs of younger people (Kershaw et al. 2017). Such a disconnect—between policy and what matters to youth—may lead to feelings of alienation, indifference and despair among some young people. Perceptions of worsening economic and social life coupled with feelings of political abandonment may have downstream negative repercussions on young people's sense of political efficacy, overall trust in government, and participation in the democratic process (Harris et al. 2010). In failing to act against precarity, governments and policy makers risk further reducing young people's sense of citizenship.

While young people were perceived as less politically active than their older counterparts (Furlong and Cartmel 2012), a past study found that young people did express interest in politics itself, but were critical of those elected to positions of power (Henn et al. 2002). Young people have reported feelings of ‘betrayal’ regarding positive government responses to climate change (Hickman et al. 2021). Despite having contributed the least to climate change, younger generations in this study and others have acknowledged that it will impact them the most in the future (Galway and Field 2023). In studies of ‘eco‐reproductive concerns’, young people expressed doubt about the wellbeing of hypothetical children in a future where climate disasters are the norm (Smith et al. 2023).

Our study is in line with research highlighting the negative impact of various forms of precarity on young people's mental health, including financial instability, precarious employment and unstable living conditions. Experiences of unaffordable housing have increased over time (Martínez del Olmo 2024) and now disproportionately affect young people in the rental sector (Arundel et al. 2024). Young people have reported frustration with employment, but with a focus on employers' unrealistic expectations for past work experience (Moore 2019) and how underemployment is compounded by other forms of precarity (Walker et al. 2016). Today, more young adults live with their parents compared to the past (Srinivas 2019), and some participants mentioned living with their parents to manage costs—financial difficulties contribute to modern trends of parental co‐residence.

Increasing precarity partially explains the mounting pressures which young people are experiencing—a previous study reported a linear increase in perceived parental expectations on university students between 1989 and 2019, and related this to a rise in ‘perfectionism’ among young people compared to the past (Curran and Hill 2022). Our study extends these findings by highlighting the perceived intergenerational injustice of precarious circumstances, pointing to the negative impact of trends that have shaped social and economic life since the global financial crisis and the COVID‐19 pandemic. In our study, participants perceived these trends as having affected younger people to a greater extent, in part due to the perceived failure of successive governments to adequately support younger generations through these crises. Policy shifts in favour of young people's social and economic conditions are needed.

Certain facets of modern life perpetuate intergenerational injustice as they exert pressures on young people which older generations did not have to contend with. Social media use can have a negative impact on mental health and is likely to contribute to psychological distress (Gupta et al. 2022). Many participants in our study agreed. Specifically, participants mentioned that social media heightens young people's expectations of each other, and while research into this effect is ongoing, studies have identified links between social media use and idealisation of a successful lifestyle (Athifah and Lilawati 2023). In our study and others, young people raised this as a negative consequence of increased digitisation (Bergoč and Novak 2024).

Participants described how intergenerational inequities had a significant impact on their mental health, with many participants feeling deep anxiety over their lives and futures. These findings are consistent with claims set forth in the Lancet Commission (McGorry et al. 2024) and policy documents that such injustice may be exerting far‐reaching negative effects on young people's health and wellbeing. The types of mental health problems reported were representative of common forms of mental distress, such as anxiety and depression. Transdiagnostic clinical frameworks have highlighted how low symptoms such as anxiety and depression can have a significant impact on young people's daily functioning and can serve as risk factors for less common and oftentimes more disabling problems such as psychosis (Shah et al. 2020). Addressing intergenerational injustice can therefore support broader efforts to prevent common types of mental health problems from emerging and potentially becoming more disabling.

Finally, our findings have implications for prevention, clinical practice and service development. With respect to prevention, these findings suggest that efforts to prevent the development of mental health challenges should target the upstream factors presented in our paper, as these are of relevance for young people. With regards to practice, clinicians would benefit from greater awareness of the importance of intergenerational injustice in the lives of young people. Doing so may enhance therapeutic relationships and potentially improve clinical outcomes. Finally, more services staffed by multidisciplinary teams are needed, such as in Integrated Youth Services (McGorry et al. 2024), as these cater to a broad range of psychosocial needs beyond symptoms. Such service models are needed to address the complex, multidimensional economic and social needs that intergenerational injustice may drive among youth seeking help.

### 5.1 Strengths and Limitations

We ensured congruence between our epistemological and ontological stance, overall methods, data collection and analysis, which is crucial for ensuring rigour in qualitative research. Our sample included people from diverse ethnic and racial backgrounds, allowing us to understand the lived experiences of people typically underrepresented in research, which likely allowed us to understand how discrimination may be an intergenerational issue for racialised youth. Limitations of the study include not recruiting participants from a well‐characterised setting, which may have resulted in heterogeneity among participants' perspectives. We recruited participants who explicitly felt as though intergenerational injustice impacted their lives; hence, our findings cannot be generalised to broader samples of youth, such as youth with different degrees of privilege. However, our recruitment method was the most feasible given the time constraints of the project, and our aim to understand experiences around intergenerational injustice, mental health and belonging more broadly.

### 5.2 Future Directions

Additional qualitative research is needed to understand how intergenerational injustice is perceived among policymakers, government, and service providers. Future quantitative research is needed to determine the generalisability of our findings to a larger subset of young people, and to understand how youth experiencing multiple forms of marginalisation (e.g., racism, classism, etc.) feel impacted by intergenerational inequities vis‐à‐vis their mental health. In this respect, intergenerational injustice can be a useful framework to evaluate against other forms of marginalisation within intersectional analyses.

## Funding

The authors have nothing to report.

## Ethics Statement

Ethical approval for the study was granted by the University of Birmingham Science, Technology, Engineering and Mathematics Ethical Review Committee (ERN_1030‐Sep2023).

## Consent

Prior to participating, participants read information sheets, signed consent forms, and were informed of UK‐based mental health support services for additional support.

## Conflicts of Interest

The authors declare no conflicts of interest.

## Data Availability

Data are not available.

## References

[eip70273-bib-0001] Alidoust, S. , and W. Huang . 2023. “A Decade of Research on Housing and Health: A Systematic Literature Review.” Reviews on Environmental Health 38, no. 1: 45–64. 10.1515/reveh-2021-0121. 34751014

[eip70273-bib-0002] Armocida, B. , L. Monasta , S. Sawyer , et al. 2022. “Burden of Non‐Communicable Diseases Among Adolescents Aged 10‐24 Years in the EU, 1990‐2019: A Systematic Analysis of the Global Burden of Diseases Study 2019.” Lancet Child & Adolescent Health 6, no. 6: 367–383. 10.1016/S2352-4642(22)00073-6. PMC9090900 35339209

[eip70273-bib-0003] Arnett, J. J. 2000. “Emerging Adulthood. A Theory of Development From the Late Teens Through the Twenties.” American Psychologist 55, no. 5: 469–480. 10842426

[eip70273-bib-0004] Arundel, R. , L. Ang , B. Emma , and R. Bentley . 2024. “Housing Unaffordability and Mental Health: Dynamics Across Age and Tenure.” International Journal of Housing Policy 24, no. 1: 44–74. 10.1080/19491247.2022.2106541.

[eip70273-bib-0005] Athifah, C. , and K. Lilawati . 2023. “Hustle Culture in Social Media: Exploring the Imagined Success in the Modern Era.” Athena: Journal of Social, Culture and Society 1, no. 4: 180–191. 10.58905/athena.v1i4.151.

[eip70273-bib-0006] Bergoč, Z. , and V. Novak . 2024. “Young People's View on the Impact of Digitalisation and Artificial Intelligence on Modern Work Practice.” Int J Econ Law 14, no. 42: 11–25.

[eip70273-bib-0007] Braun, V. , and V. Clarke . 2021. Thematic Analysis: A Practical Guide. SAGE Publications Ltd. http://digital.casalini.it/9781526417305.

[eip70273-bib-0008] Caney, S. 2019. “Democratic Reform, Intergenerational Justice and the Challenges of the Long‐Term. CUSP Essay Series on the Morality of Sustainable Prosperity.” 11, 3–7.

[eip70273-bib-0009] Christophers, B. 2018. “Intergenerational Inequality? Labour, Capital, and Housing Through the Ages.” Antipode 50, no. 1: 101–121. 10.1111/anti.12339.

[eip70273-bib-0010] Curran, T. , and A. P. Hill . 2022. “Young People's Perceptions of Their Parents' Expectations and Criticism Are Increasing Over Time: Implications for Perfectionism.” Psychological Bulletin 148, no. 1–2: 107–128. 10.1037/bul0000347. 35357848

[eip70273-bib-0011] Furlong, A. , and F. Cartmel . 2012. “Social Change and Political Engagement Among Young People: Generation and the 2009/2010 British Election Survey.” Parliamentary Affairs 65, no. 1: 13–28. 10.1093/pa/gsr045.

[eip70273-bib-0012] Future Generations Act . 2015. “Future Generations Act.” https://futuregenerations.wales/discover/about‐future‐generations‐commissioner/future‐generations‐act‐2015/.

[eip70273-bib-0013] Galasso, V. , A. E. Galeotti , A. Bellia , et al. 2025. “An Index of Intergenerational Justice: Main Concepts and Preliminary Evidence From the Age‐It Research Program.” Journals of Gerontology. Series B, Psychological Sciences and Social Sciences 80: S169–S179. 10.1093/geronb/gbaf192. PMC12736992 41442761

[eip70273-bib-0014] Galway, L. P. , and E. Field . 2023. “Climate Emotions and Anxiety Among Young People in Canada: A National Survey and Call to Action.” Journal of Climate Change and Health 9: 100204. 10.1016/j.joclim.2023.100204.

[eip70273-bib-0015] Guba, E. G. , and Y. S. Lincoln . 2005. “Paradigmatic Controversies, Contradictions, and Emerging Confluences.” In The Sage Handbook of Qualitative Research, 3rd ed., 191–215. Sage Publications Ltd.

[eip70273-bib-0016] Gupta, C. , D. S. Jogdand , and M. Kumar . 2022. “Reviewing the Impact of Social Media on the Mental Health of Adolescents and Young Adults.” Cureus 14, no. 10: e30143. 10.7759/cureus.30143. PMC9645642 36381882

[eip70273-bib-0017] Harris, A. , J. Wyn , and S. Younes . 2010. “Beyond Apathetic or Activist Youth: ‘Ordinary’ Young People and Contemporary Forms of Participation.” Young 18, no. 1: 9–32. 10.1177/110330880901800103.

[eip70273-bib-0018] Henn, M. , M. Weinstein , and D. Wring . 2002. “A Generation Apart? Youth and Political Participation in Britain.” British Journal of Politics and International Relations 4, no. 2: 167–192.

[eip70273-bib-0019] Hickman, C. , E. Marks , P. Pihkala , et al. 2021. “Climate Anxiety in Children and Young People and Their Beliefs About Government Responses to Climate Change: A Global Survey.” Lancet Planetary Health 5, no. 12: e863–e873. 10.1016/S2542-5196(21)00278-3. 34895496

[eip70273-bib-0020] Jordan, G. , F. Akhtar , N. Ahsan , et al. 2024. “Intergenerational Injustice and Youth Mental Health: A Call to Action.” Early Intervention in Psychiatry 18, no. 11: 897–900. 10.1111/eip.13619. 39391960

[eip70273-bib-0021] Kershaw, P. , E. Swanson , and A. Stucchi . 2017. “A Surgical Intervention for the Body Politic: Generation Squeeze Applies the Advocacy Coalition Framework to Social Determinants of Health Knowledge Translation.” Canadian Journal of Public Health 108, no. 2: e199–e204. 10.17269/CJPH.108.5881. PMC6972439 28621657

[eip70273-bib-0022] Malterud, K. , V. D. Siersma , and A. D. Guassora . 2015. “Sample Size in Qualitative Interview Studies: Guided by Information Power.” Qualitative Health Research 26, no. 13: 1753–1760. 10.1177/1049732315617444. 26613970

[eip70273-bib-0023] Martin, G. 2024. “Climate Change and Mental Health and Wellbeing: Reflections From a Health Geography Lens.” Geography Compass 18, no. 1: e12734. 10.1111/gec3.12734.

[eip70273-bib-0024] Martínez del Olmo, A. 2024. “Less Access and More Inequality: Evidence of and Responses to the Housing Affordability Crisis Faced by Young People in Spain's Larger Cities.” Critical Housing Analysis 2: 229–241.

[eip70273-bib-0025] McGorry, P. D. , C. Mei , N. Dalal , et al. 2024. “The Lancet Psychiatry Commission on Youth Mental Health.” Lancet Psychiatry 11, no. 9: 731–774. 10.1016/s2215-0366(24)00163-9. 39147461

[eip70273-bib-0026] Moore, K. 2019. “The Quest for Sustainable Employment: Challenges Faced by Young People During the Job‐Search Process.” Australian Journal of Social Issues 54, no. 1: 91–108. 10.1002/ajs4.60.

[eip70273-bib-0027] Orben, A. , A. Meier , T. Dalgleish , and S.‐J. Blakemore . 2024. “Mechanisms Linking Social Media Use to Adolescent Mental Health Vulnerability.” Nature Reviews Psychology 3, no. 6: 407–423. 10.1038/s44159-024-00307-y.

[eip70273-bib-0028] Patel, P. K. , L. D. Leathem , D. L. Currin , and K. H. Karlsgodt . 2021. “Adolescent Neurodevelopment and Vulnerability to Psychosis.” Biological Psychiatry 89, no. 2: 184–193. 10.1016/j.biopsych.2020.06.028. PMC9397132 32896384

[eip70273-bib-0029] Pickard, S. 2019. “Age War as the New Class War? Contemporary Representations of Intergenerational Inequity.” Journal of Social Policy 48, no. 2: 369–386. 10.1017/S0047279418000521.

[eip70273-bib-0030] Puig‐Barrachina, V. , C. Vanroelen , A. Vives , et al. 2014. “Measuring Employment Precariousness in the European Working Conditions Survey: The Social Distribution in Europe.” Work 49, no. 1: 143–161. 10.3233/WOR-131645 23803435

[eip70273-bib-0031] Rawls, J. 2001. Justice as Fairness: A Restatement. Harvard University Press.

[eip70273-bib-0032] Reinharz, S. 1997. “Who Am I? The Need for a Variety of Selves in the Field.” In Reflexivity and Voice, 3–20. SAGE.

[eip70273-bib-0033] Sandelowski, M. , J. Barroso , and C. I. Voils . 2007. “Using Qualitative Metasummary to Synthesize Qualitative and Quantitative Descriptive Findings.” Research in Nursing & Health 30, no. 1: 99–111. 10.1002/nur.20176. PMC2329806 17243111

[eip70273-bib-0034] Shah, J. L. , J. Scott , P. D. McGorry , et al. 2020. “Transdiagnostic Clinical Staging in Youth Mental Health: A First International Consensus Statement.” World Psychiatry 19, no. 2: 233–242. 10.1002/wps.20745. PMC7215079 32394576

[eip70273-bib-0035] Sinha, G. R. , V. Madhubalan , and C. R. Larrison . 2024. “Student Loan Debt and Mental Health: A Comprehensive Review of Scholarly Literature From 1900 to 2019.” Journal of Evidence‐Based Social Work 21, no. 3: 363–393. 10.1080/26408066.2023.2299019. 38179674

[eip70273-bib-0036] Sisk, L. M. , and D. G. Gee . 2025. “Developmental Neuroplasticity and Adversity‐Related Risk for Psychopathology.” Neuropsychopharmacol 50, no. 1: 316–317. 10.1038/s41386-024-01950-y. PMC11525816 39095522

[eip70273-bib-0037] Smith, D. M. , J. Sales , A. Williams , and S. Munro . 2023. “Pregnancy Intentions of Young Women in Canada in the Era of Climate Change: A Qualitative Auto‐Photography Study.” BMC Public Health 23, no. 1: 766. 10.1186/s12889-023-15674-z. PMC10127979 37098525

[eip70273-bib-0038] Solmi, M. , J. Radua , M. Olivola , et al. 2022. “Age at Onset of Mental Disorders Worldwide: Large‐Scale Meta‐Analysis of 192 Epidemiological Studies.” Molecular Psychiatry 27, no. 1: 281–295. 10.1038/s41380-021-01161-7. PMC8960395 34079068

[eip70273-bib-0039] Srinivas, V. 2019. “Explaining the Increase in Young Adults Living With Parents.” Journal of Economic Issues 53, no. 4: 1017–1028. 10.1080/00213624.2019.1664237.

[eip70273-bib-0040] Walker, R. , L. Brown , M. Moskos , et al. 2016. “‘They Really Get You Motivated’: Experiences of a Life‐First Employment Programme From the Perspective of Long‐Term Unemployed Australians.” Journal of Social Policy 45, no. 3: 507–526. 10.1017/S0047279416000027.

[eip70273-bib-0041] Wolf, K. , and J. Schmitz . 2024. “Scoping Review: Longitudinal Effects of the COVID‐19 Pandemic on Child and Adolescent Mental Health.” European Child & Adolescent Psychiatry 33, no. 5: 1257–1312. 10.1007/s00787-023-02206-8. PMC10119016 37081139

